# Control of cell death and mitochondrial fission by ERK1/2 MAP kinase signalling

**DOI:** 10.1111/febs.14122

**Published:** 2017-06-18

**Authors:** Simon J. Cook, Kate Stuart, Rebecca Gilley, Matthew J. Sale

**Affiliations:** ^1^ Signalling Programme The Babraham Institute Cambridge UK

**Keywords:** apoptosis, BCL2, BH3‐only proteins, BIM, BRAF, DRP1, ERK1/2, MEK1/2, mitochondria, RAS

## Abstract

The ERK1/2 signalling pathway is best known for its role in connecting activated growth factor receptors to changes in gene expression due to activated ERK1/2 entering the nucleus and phosphorylating transcription factors. However, active ERK1/2 also translocate to a variety of other organelles including the endoplasmic reticulum, endosomes, golgi and mitochondria to access specific substrates and influence cell physiology. In this article, we review two aspects of ERK1/2 signalling at the mitochondria that are involved in regulating cell fate decisions. First, we describe the prominent role of ERK1/2 in controlling the BCL2‐regulated, cell‐intrinsic apoptotic pathway. In most cases ERK1/2 signalling promotes cell survival by activating prosurvival BCL2 proteins (BCL2, BCL‐x_L_ and MCL1) and repressing prodeath proteins (BAD, BIM, BMF and PUMA). This prosurvival signalling is co‐opted by oncogenes to confer cancer cell‐specific survival advantages and we describe how this information has been used to develop new drug combinations. However, ERK1/2 can also drive the expression of the prodeath protein NOXA to control ‘autophagy or apoptosis’ decisions during nutrient starvation. We also describe recent studies demonstrating a link between ERK1/2 signalling, DRP1 and the mitochondrial fission machinery and how this may influence metabolic reprogramming during tumorigenesis and stem cell reprogramming. With advances in subcellular proteomics it is likely that new roles for ERK1/2, and new substrates, remain to be discovered at the mitochondria and other organelles.

**Abbreviations**
A1: BCL2‐related protein A1
AP1: activator protein 1
APAF1: apoptotic peptidase‐activating factor 1
ARAF: v‐raf murine sarcoma 3611 viral oncogene homologue
BAD: BCL‐XL/BCL2‐associated death promoter
BAK: BCL2 homologous antagonist/killer
BAX: BCL2‐associated x protein
BCL2: B‐cell lymphoma 2
BCL‐XL: B‐cell lymphoma extra large
BH3: BCL2 homology domain 3
BID: BH3‐interacting domain death agonist
BIK: BCL2‐interacting killer
BIM: BCL2‐interacting mediator of cell death
BMF: BCL2‐modifying factor
BOP: BH3‐only protein
BRAF: v‐raf murine sarcoma viral oncogene homologue B1
CDK: cyclin‐dependent kinase
CRAF: v‐raf‐1 murine leukaemia viral oncogene homologue 1
CREB: cAMP‐responsive element‐binding protein
CRC: colorectal cancer
DKO: double knockout
DRP1: dynamin‐related GTPase protein 1
DUSP: dual‐specificity phosphatase
E1A: Adenovirus early region 1A
ELK1: ETS‐like gene 1
ERK: extracellular signal‐regulated kinase
ES: embryonic stem cell
Fis1: mitochondrial fission protein 1
FOXO3: forkhead box 3
G1: growth phase 1
GSK3: glycogen synthase kinase 3
HEK: human embryonic kidney
HRAS: v‐Ha‐ras Harvey rat sarcoma viral oncogene homologue
Hrk: harakiri [BCL2 interacting protein (contains only BH3 domain)]
hTERT: human telomerase reverse transcriptase
iPSC: induced pluripotent stem cell
JNK: c‐Jun N‐terminal Kinase
Klf4: Kruppel‐like factor 4
KRAS: v‐Ki‐ras2 Kirsten rat sarcoma viral oncogene homologue
MCL1: myeloid cell leukaemia 1
MDM2: Mouse double minute 2 homolog [also known as p53 E3 ubiquitin ligase homologue (mouse)]
MEK: MAPK or ERK kinase
Mff: mitochondrial fission factor
Mfn: mitofusin
MOMP: mitochondrial outer membrane permeabilisation
MSK: mitogen‐ and stress‐activated protein kinase
MYC: v‐myc myelocytomatosis viral oncogene homologue (avian)
NRAS: neuroblastoma RAS viral (v‐ras) oncogene homologue
Oct4: octamer‐binding transcription factor 4
OMM: outer mitochondrial membrane
Opa1: optic atrophy 1
PI3K: phosphoinositide 3‐kinase
PUMA: p53‐up‐regulated modulator of apoptosis
RAS: rat sarcoma virus oncogene
RNAi: RNA interference
RSK: ribosomal protein S6 kinase
Sox2: Sex determining region Y‐box 2
SV40: Simian virus 40
VDAC: voltage‐dependent anion channel

## Introduction

Lifelong health requires that cells are able to sense and adapt to changes in their environment. Cells of the embryo must respond to specific cues and make the correct developmental decisions, which may involve cell division, cell cycle arrest and differentiation into specific cell lineages, or cell death. In the adult, cells are constantly exposed to damage or stress, including DNA damage, and must mount appropriate responses (survival, senescence, death) to maintain genomic and organismal integrity. Responses to environmental cues are initiated and controlled by the activation of signal transduction pathways that involve protein kinases, enzymes that catalyse the post‐translational phosphorylation of specific protein substrates. Since protein kinase signalling pathways control fundamental cell fate decisions they are subject to very fine control, including negative feedback loops 1 and the action of protein phosphatases 2 to ensure measured and appropriate responses. Loss of control results in deregulated signalling, which can drive disease; indeed, protein kinases are the most frequently mutated class of genes in human cancer 3, where they drive inappropriate proliferation and survival, some of the hallmarks of cancer 4, 5.

The RAS‐regulated RAF‐MEK‐ERK signalling pathway is one of the best understood signalling pathways and plays a key role in controlling cell proliferation, differentiation and cell survival or cell death decisions 6, 7. In this pathway, growth and survival factors activate the RAS GTPases by promoting GDP release to allow GTP binding. Active RAS‐GTP then binds to one of the RAF protein kinases (A, B or CRAF) resulting in their activation. RAF phosphorylates and activates MEK1 and MEK2 (MAPK or ERK kinase), which in turn phosphorylate and activate ERK1 and ERK2 (extracellular signal‐regulated kinases). Active ERK1/2 then phosphorylate > 200 substrates 8, 9 including other protein kinases, transcription factors, RNA‐binding proteins, regulators of mRNA translation and regulators of cell death. The consequences of ERK1/2 activation are many and varied; indeed, ERK1/2 activation can promote apparently contradictory biological responses such as cell cycle progression and cell cycle arrest; cell survival or cell death. Specific responses are determined by a variety of factors including the duration and magnitude of ERK1/2 activation, the subcellular distribution of ERK1/2 (which allows or limits access to specific substrates) and the coincident activation of other pathways 8, 9.

Quite apart from its role in normal biology (including development) the ERK1/2 pathway is strongly implicated in cancer 10, 11 and our appreciation of its role in cell death/survival signalling has emerged, in part, from studies in cancer cells where the pathway is deregulated. Components of the pathway, including growth factor receptors, regulators of RAS, the RAS proteins themselves, BRAF, MEK1 and MEK2 are mutated in a variety of cancers, resulting in hyperactivation of ERK1/2 signalling which underpins the growth and maintenance of many tumour types. Indeed, certain tumours are notable for their high incidence of activating mutations in KRAS (pancreatic, colorectal and nonsmall cell lung cancer) and BRAF (melanoma, thyroid, hairy cell leukaemia); such cancer cells typically become addicted to these oncoproteins and the hyperactivation of ERK1/2 signalling for proliferation and survival, making the pathway an attractive target for therapeutic intervention. For example, inhibitors of BRAF or MEK1/2 have now been approved for the treatment of melanoma harbouring the common BRAF^V600E^ mutation 10, 11. As a result of many drug discovery programmes there are now a wealth of highly selective RAF inhibitors, specific allosteric MEK1/2 inhibitors and a growing number of selective ERK1/2 inhibitors. These inhibitors, together with constitutively active or conditionally active mutants of RAF or MEK, serve as excellent research tools for probing the role of the ERK1/2 pathway.

The ERK1/2 signalling pathway is best known for its role in connecting activated growth factor receptors to changes in gene expression by virtue of the ability of ERK1/2 to translocate into the nucleus and phosphorylate transcription factors. However, there is a growing appreciation that active ERK1/2 also translocate to a variety of other organelles including the endoplasmic reticulum, endosomes/lysosomes, golgi and mitochondria to access specific substrates and thereby influence cell physiology 12, 13. For example, ERK1/2 can rapidly translocate to mitochondria and associate with specific mitochondrial proteins 14 to influence metabolism 15, mitophagy, in which defective, depolarised mitochondria are targeted to the autophagolysosome for recycling by autophagy 16 and apoptosis through the regulation of BCL2 proteins 7. In this article, we review two aspects of ERK1/2 signalling at the mitochondria. First, we describe how ERK1/2 signalling controls the BCL2‐regulated, cell‐intrinsic apoptotic pathway and how this knowledge has recently been used to develop new drug combinations for the treatment of cancer. We also review recent studies demonstrating a link between ERK1/2 signalling and the mitochondrial fission machinery and how this may influence metabolic reprogramming during tumorigenesis and stem cell reprogramming.

### The BCL2‐regulated cell‐intrinsic pathway of apoptosis

**Figure 1:**
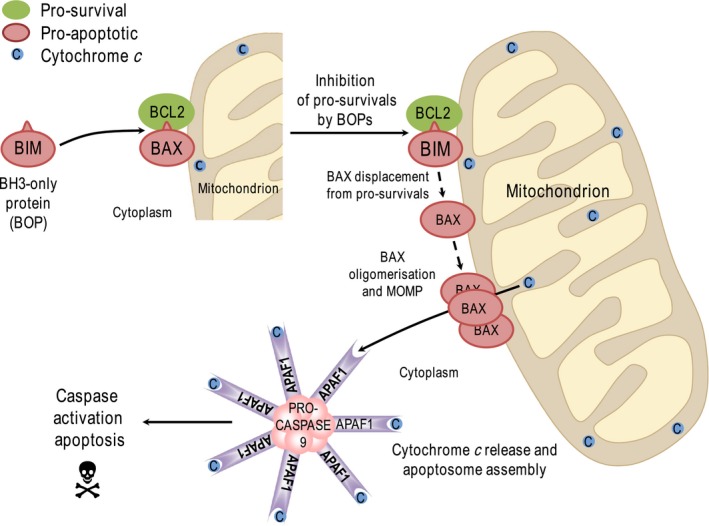
Overview of the cell‐intrinsic pathway of apoptosis. The cell‐intrinsic or mitochondrial pathway of apoptosis is regulated by the BCL2 protein family. Prosurvival proteins represented here by BCL2 (but also including BCL‐W, BCL‐XL, MCL1 and A1) bind to and inhibit the proapoptotic effector proteins BAX and BAK. BH3‐only proteins (BOPs), represented here by BIM, are upregulated or activated in response to various stresses (growth factor withdrawal, DNA damage, etc). All BOPs can bind to and inhibit a subset of the prosurvival proteins, thereby releasing BAX and BAK to undergo further activation steps including oligomerisation in the outer mitochondrial membrane; this results in mitochondrial outer membrane permeabilisation (MOMP) and release of cytochrome *c* from the intermembrane space. Cytochrome *c* binds to APAF1 inducing conformational changes that result in the assembly of the heptameric apoptosome, which serves as a platform for activation of the initiator caspase, caspase‐9. Caspase‐9 then cleaves effector caspases such as caspase‐3 and caspase‐7, which in turn cleave a large number of proteins within the cell to drive apoptosis. Note that some BOPs (‘activators’, such as BIM and BID), may also bind directly to BAX/BAK to promote their activation. Other BOPs (such as BAD or BIK) are not strong activators of BAX/BAK but can liberate activator BOPs from prosurvival protein‐mediated repression. See text for details.

The BCL2‐regulated, cell‐intrinsic pathway of apoptosis is an evolutionarily conserved cell death pathway that underpins developmental decisions as well as responses to stress and damage 17. Proapoptotic signals drive mitochondrial outer membrane permeabilisation (MOMP), releasing cytochrome *c* from the intermembrane space into the cytosol where it binds to APAF1, promoting assembly of the apoptosome and recruitment and proteolytic activation of procaspase‐9. Active caspase‐9 is then able to cleave and activate the ‘executioner’ caspases, caspase‐3 and caspase‐7, which go on to cleave a large number of cellular substrates, ultimately resulting in apoptosis 18. This cell‐intrinsic apoptotic pathway is controlled by interactions between members of the BCL2 protein family, which control the integrity of the outer mitochondrial membrane (OMM) 19 (Fig. 1).

The BCL2 protein family consists of antagonistic prodeath and prosurvival proteins. The prosurvival proteins (BCL2, BCL‐w, BCL‐XL, MCL1 and A1) contain four BCL2‐homology domains (BH1–4) and reside on the OMM where they inhibit apoptosis by binding to prodeath proteins. The prodeath proteins comprise the key death effector proteins (BAX and BAK) and the BH3‐only proteins (or BOPs) including BAD, BID, BIK, BIM, BMF, HRK, NOXA and PUMA; the BOPs are a structurally diverse group of proteins that share in common only their BH3 domain. In the absence of stress the prosurvival proteins bind and inhibit BAX and BAK, preventing them from undergoing homo‐oligomerisation, which otherwise leads to MOMP 19. Following different forms of stress or damage, specific BOPs are activated or expressed and bind to and inhibit prosurvival proteins, allowing BAX or BAK to drive MOMP and apoptosis (Fig. 1). Most BOPs only target a subset of prosurvival proteins but BIM and PUMA are able to target all prosurvival proteins, and this likely explains their superior potency. Additionally, some BH3‐only proteins (e.g. BIM, BID, PUMA) may directly activate BAX and BAK 19, 20.

While stress‐induced signalling can regulate the core death effector proteins BAX or BAK, for example, by p53‐dependent expression of BAX 21, most stress or survival signalling appears to engage the pathway through regulation of the prosurvival proteins or the BOPs. Indeed, the structural diversity of the BOPs, outside of their BH3 domain, allows them to be targeted by different prodeath or prosurvival signalling pathways. For example, phosphorylation of Ser136 within BAD, targeted by the phosphoinositide 3′‐kinase (PI3K)‐dependent prosurvival kinase protein kinase B (PKB/AKT), allows it to be sequestered away from the mitochondria by 14‐3‐3 proteins 22, 23. Similarly, BIM and BMF have binding sites for dynein light chain‐1 (DLC1) or DLC2 allowing them to be sequestered away from mitochondria to the microtubule network or actin cytoskeleton respectively 24, 25; this in turn allows them to be mobilized from these ‘reservoirs’ in response to disruption of the actin cytoskeleton, microtubule network or phosphorylation by JNK 26. Several BOPs have been proposed to be intrinsically unstructured proteins (IUPs) that are disordered in the absence of binding partners 27. Disorder tends to favour rapid interactions with multiple partner proteins. In addition, protein kinase binding appears to be facilitated by disorder; certainly protein phosphorylation occurs predominantly within disordered regions 28. Finally, proteasomal degradation appears to be more efficient for proteins with disordered initiation sites 29. Together, these observations suggest that unstructured regions within BOPs may confer regulation by a variety of signalling mechanisms.

The ERK1/2 signalling cascade has emerged as a principal regulator of the BCL2 protein family and cell‐intrinsic pathways of apoptosis. This is achieved by influencing the expression and/or activity of many members of the BCL2 protein family, the mechanistic bases for which are discussed below.

#### Regulation of prosurvival BCL2 proteins by the ERK1/2 pathway

**Figure 2:**
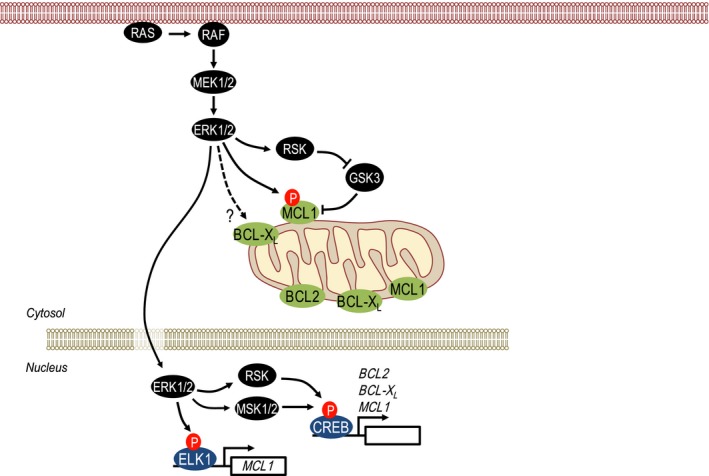
Regulation of prosurvival BCL2 proteins by ERK1/2 signalling. The expression of prosurvival BCL2 proteins is subject to multiple levels of regulation by ERK1/2 signalling. Direct phosphorylation of MCL1 by ERK1/2 stabilizes MCL1, whereas GSK3‐mediated phosphorylation promotes MCL1 degradation but can be countered by the ERK1/2‐dependent, RSK‐catalysed phosphorylation and inhibition of GSK3. In the nucleus, ERK1/2 influences the transcription of multiple BCL2 family members. Activation of ERK1/2‐dependent RSK and MSK1/2 activates CREB, which promotes transcription of *BCL2, BCL‐* *X*_*L*_ and *MCL1*. ERK1/2 can also drive transcription of *MCL1* via phosphorylation of the ETS family transcription factor ELK1. See the text for details.

Several prosurvival BCL2 proteins are regulated at the level of transcription including BCL2, BCL‐X_L_ and MCL1 (Fig. 2). For example, cAMP‐responsive element‐binding protein (CREB) is known to promote transcription of *BCL2*,* BCL‐X*_*L*_ and *MCL1* in response to ERK1/2 signalling 30, 31, 32 and this most likely reflects ERK1/2‐dependent phosphorylation and activation of the kinases RSK and MSK, which can phosphorylate and activate CREB 33. In addition, *MCL1* transcription is also promoted by ERK1/2‐mediated phosphorylation and activation of the transcription factor ELK1, which binds to the *MCL1* promoter 34, 35, 36.

The MCL1 protein has a short half‐life that is regulated by phosphorylation of sites within its PEST domain, a peptide sequence rich in proline (P), glutamic acid (E), serine (S) and threonine (T). ERK1/2 directly phosphorylate Thr163 within this domain to stabilize MCL1 37, whereas GSK3‐catalysed phosphorylation within the PEST domain promotes MCL1 turnover 38. Thus, ERK1/2 signalling drives dual regulation of MCL1 stability: direct phosphorylation by ERK1/2 and inactivation of GSK3 by the ERK1/2‐dependent RSK kinases both acting to promote MCL1 stability (Fig. 2). In addition, BCL‐X_L_ is also regulated at the level of protein stability; certainly activated mutant RAS proteins are able to increase BCL‐X_L_ protein stability, although this effect has not been attributed specifically to ERK1/2 signalling or indeed to direct BCL‐X_L_ phosphorylation 39. Finally, BCL2 itself is also phosphorylated by ERK1/2 at Ser87; in contrast to most other studies this phosphorylation event is proposed to inhibit its prosurvival function, thereby promoting cell death. However, phosphorylation of BCL2 at Ser87 was only reliably observed with an artificial ∆TM mutant of BCL2 that fails to associate with mitochondria; it was not observed with wild‐type BCL2, calling into question its physiological relevance 40.

#### Regulation of prodeath BH3‐only proteins by the ERK1/2 pathway

**Figure 3:**
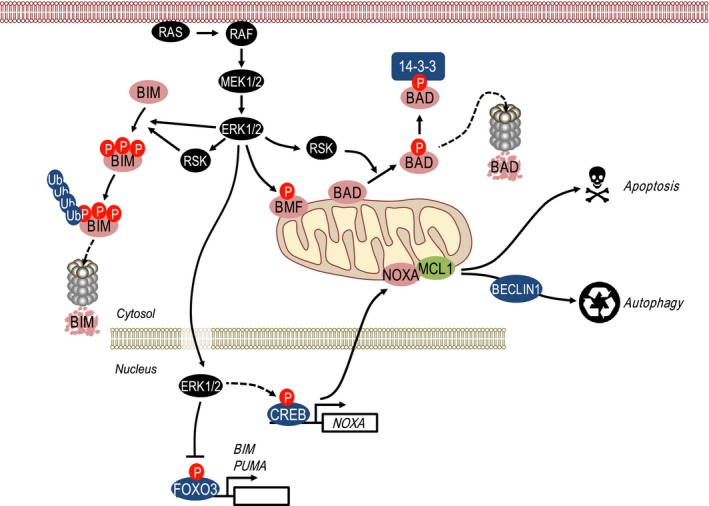
Regulation of prodeath BH3‐only proteins (BOPs) by ERK1/2 signalling. The BH3‐only protein BIM_EL_ is phosphorylated by ERK1/2 at multiple sites, marking BIM for ubiquitination and subsequent degradation by the 26S proteasome; phosphorylation by ERK1/2 may ‘prime’ BIM for phosphorylation by RSK as part of this degradation signal. ERK1/2 also negatively regulates the expression and/or activity of BMF although the underlying mechanisms remain undefined. RSK, activated by ERK1/2, promotes BAD phosphorylation, which creates a 14‐3‐3‐binding site and sequesters BAD away from the mitochondria. ERK1/2 also promotes the degradation of FOXO3, thereby inhibiting FOXO3‐dependent transcription of *BIM* and *PUMA*. Of note, in contrast to other proapoptotic BOPs, ERK1/2 signalling actually promotes the expression of NOXA mRNA and protein, which may be linked to ERK1/2‐driven autophagy. Thus, with the exception of NOXA, ERK1/2 signalling typically represses the expression and/or activity of the proapoptotic BH3‐only proteins. See the text for details.

Six of the known BOPs have been proposed to be regulated by ERK1/2 signalling by a variety of mechanisms (Fig. 3). Five of these (BAD, BIM, BMF, PUMA, BIK) are inhibited or repressed by ERK1/2 signalling while the sixth, NOXA, is expressed in response to ERK1/2 signalling and may serve to link ERK1/2 signalling to autophagy.

BCL‐XL/BCL2‐associated death promoter (BAD) is subject to multisite phosphorylation that represses its proapoptotic activity by promoting 14‐3‐3‐dependent sequestration and by disrupting BAD binding to prosurvival proteins. The first major insight into phosphorylation‐dependent BAD regulation came with demonstration that only nonphosphorylated BAD could bind to BCL‐X_L_, the identification of Ser112 and Ser136 as phosphorylation sites and the recognition that both sites conformed to a RXRXXS 14‐3‐3‐binding motif 22. Mutagenesis demonstrated that phosphorylation at either Ser112 or Ser136 was independently capable of conferring 14‐3‐3 binding, whereas a Ser112Ala/Ser136Ala double mutant failed to bind 14‐3‐3. This led to the proposal that phosphorylation‐dependent binding to 14‐3‐3 proteins sequestered BAD in the cytosol away from prosurvival BCL2 proteins at the mitochondria. Subsequent studies showed that growth factor‐stimulated phosphorylation of Ser112 was inhibited by MEK inhibitors 41, 42, whereas Ser136 phosphorylation was inhibited by PI3K inhibitors 33, 43. The ERK‐dependent kinase RSK was subsequently shown to phosphorylate Ser112 44 while PKB/AKT was shown to phosphorylate Ser136 23, 43. Since either Ser112 or Ser136 phosphorylation can independently confer 14‐3‐3 binding 22 it appears that ERK‐RSK and PI3K‐PKB serve as parallel redundant inputs to repress the apoptotic activity of BAD. In addition, these phosphorylation events appear to orchestrate phosphorylation of Ser155 within the BAD BH3 domain by cAMP‐dependent protein kinase A (PKA), thereby directly blocking BAD binding to BCL‐X_L_; for example, 14‐3‐3 binding to BAD (which is dependent on Ser112 or Ser136 phosphorylation) has been shown to promote PKA‐dependent phosphorylation at Ser155 45. Thus, ERK‐RSK or PI3K‐PKB signalling may serve in a hierarchy with cAMP‐PKA to inactivate BAD. Finally, MSK1, a RSK‐related protein kinase that is activated by ERK1/2 or p38‐dependent phosphorylation, can also promote BAD phosphorylation at Ser112, providing a further ERK1/2‐dependent input to repress BAD‐induced apoptosis 46.

More recently at least two studies have suggested that ERK1/2 activation promotes the proteasome‐dependent turnover of BAD 47, 48. Furthermore, one of these studies suggested that RSK‐dependent phosphorylation at Ser112 is required for this proteasome‐dependent turnover 48. This ERK1/2‐induced turnover of BAD may be cell type specific, because a previous study of ERK1/2‐dependent BIM_EL_ turnover (see below) actually used BAD as a negative control that did not turnover in response to ERK1/2 activation 49. Regardless, it will be interesting to identify the relevant E3 ubiquitin ligase and understand how it engages with BAD that has been phosphorylated at Ser112.

B‐cell lymphoma 2‐interacting mediator of cell death (BIM), and in particular the most abundant extra long isoform, BIM_EL_, has emerged as a prominent and important target of ERK1/2 signalling. Direct phosphorylation of BIM_EL_ on at least three sites by ERK1/2 targets it for ubiquitylation and subsequent proteasome‐dependent degradation 49, 50; an observation that has since been confirmed in countless biological systems. However, the precise mechanism by which phosphorylation of BIM_EL_ leads to its ubiquitylation and turnover remains unclear. It has been proposed that ERK1/2‐catalysed phosphorylation ‘primes’ or allows subsequent phosphorylation by RSK and that these phosphorylation sites provide a binding site (a phosphodegron) for the βTrCP1/2 E3 ubiquitin ligases 51. However, a BIM_EL_ mutant lacking lysine residues is defective for ubiquitylation but still undergoes ERK1/2‐driven, proteasome‐dependent turnover in cells 52. BIM_EL_ is an intrinsically unstructured protein (IUP) and in common with other IUPs can be degraded by uncapped 20S proteasomes in the absence of poly‐ubiquitylation; indeed, BIM_EL_ degradation by isolated 20S proteasomes is prevented when it is bound to the prosurvival protein MCL1 52. One observation that might be relevant here is that phosphorylation of BIM_EL_ by ERK1/2 has also been shown to disrupt BIM_EL_:MCL1 and BIM_EL_:BCL‐X_L_ complexes, with dissociated BIM_EL_ then being more rapidly turned over 53. Thus, there may be at least two pathways for degradation of this potent proapoptotic BH3‐only protein: a ubiquitin‐dependent pathway via the canonical 26S proteasome and a default ubiquitin‐independent pathway via the 20S proteasome, although how nonubiquitylated BIM_EL_ is chaperoned to 20S proteasomes remains unclear.

While βTrCP1/2 can serve as E3 ubiquitin ligases for BIM_EL_, a recent study has suggested that BIM_EL_ ubiquitylation is countered by USP27x, a deubiquitylating enzyme (DUB) 54. USP27x was found to bind to exon 3 of BIM_EL_ (the region including the ERK docking domain and ERK and RSK phosphorylation sites) but not to bind to other BIM isoforms. Furthermore, like βTrCP1/2, the binding of USP27x to BIM_EL_ was dependent upon ERK1/2 activation. Overexpression of USP27x combined with ERK1/2 activation to promote the deubiquitylation and stabilisation of BIM_EL_ and increase caspase‐dependent apoptosis. However, the authors also found that overexpression of USP27x combined with ERK1/2 pathway inhibition to promote apoptosis. Since ERK1/2 inhibition should prevent USP27x binding, this contradictory result suggests that at least some of the effects of USP27x are independent of ERK1/2 and BIM; indeed, cell death arising from overexpression of USP27x and ERK1/2 pathway inhibition was not exclusively BIM dependent. Finally, loss of endogenous USP27x in cells expressing BRAF^V600E^ resulted in a reduction in the level of the BIM_EL_ protein. It was not possible to assess the effects of endogenous USP27x on BIM_EL_ stabilisation as there was no suitable USP27x antibody to assess overexpression or knockdown. It will be interesting to determine if USP27x is the only DUB that can de‐ubiquitylate BIM_EL_.

In addition to protein stability, *BIM* transcription is positively regulated by FOXO3 55, which is itself a target of ERK1/2; ERK1/2‐mediated phosphorylation of FOXO3 promotes its nuclear exclusion and MDM2‐dependent ubiquitylation and degradation by the proteasome, thereby repressing *BIM* transcription 56 (Fig. 3). ERK1/2 was shown to phosphorylate FOXO3A directly, predominantly at Ser294, Ser344 and Ser425; indeed, these are all S/T‐P sites, the minimum motif required for phosphorylation by ERK1/2. This contrasts with the PKB/AKT‐dependent phosphorylation of FOXO3A at Thr32, Ser253 or S315 which are not proline directed. Two of these sites, Thr32 and S253, seem especially important for phospho‐dependent binding to 14‐3‐3 which sequesters FOXO3A in the cytoplasm so that it is unable to transcribe target genes in the nucleus 57. As with BAD, it appears that ERK1/2 and PI3K‐PKB serve as parallel redundant inputs to repress the activity of FOXO3A, thereby blocking BIM transcription. Thus, ERK1/2 activation represses expression of BIM_EL_ protein and mRNA and impairs its proapoptotic activity by promoting dissociation from prosurvival proteins. Indeed, tumour cells with *BRAF* mutations are addicted to ERK1/2 signalling for repression of BIM 58 and pharmacological inhibition of ERK1/2 signalling induces strong increases in BIM expression in many contexts. Notably, pretreatment BIM expression levels may be predictive biomarkers for tumour cell responses to some targeted kinase inhibitors 59.

Inhibition of ERK1/2 signalling also causes a striking increase in the expression of both BMF and PUMA. For example, inhibition of ERK1/2 in either *KRAS*‐ or *BRAF*‐mutant colorectal cancer cells leads to a strong upregulation of both proteins 60. The mechanisms by which ERK1/2 signalling represses BMF expression are unclear. Inhibition of ERK1/2 signalling can increase expression of *BMF* mRNA and promote BMF localisation to the cytosol but it is unclear if this represents relocation from the actin cytoskeleton or a simple increase in BMF expression 61, 62. In addition, ERK2 phosphorylates BMF directly on two sites, Ser74 and Ser77, with Ser77 phosphorylation proposed to inhibit BMF's proapoptotic activity, although quite how remains unclear 63. Inhibition of ERK1/2 signalling also frequently increases expression of PUMA 60, 64. Since FOXO3 can drive *PUMA* transcription in response to growth factor or cytokine withdrawal 65 ERK1/2‐dependent modulation of FOXO3 expression 56 may contribute to this up‐regulation of PUMA (Fig. 3).

More recently, ERK1/2 was proposed to regulate the stability of the BH3‐only protein BIK, in a manner analogous to BIM_EL_ 66. Direct phosphorylation of BIK on Thr124 by ERK1/2 was suggested to promote ubiquitylation and proteasome‐mediated degradation of BIK. Consistent with these observations, the authors demonstrated that MEK1/2 inhibition in tumour cells with *BRAF* or *RAS* mutations caused a striking up‐regulation of BIK protein. However, others have observed little or no change in BIK expression upon perturbation of ERK1/2 signalling in such tumour cells 67, 68. Subsequent studies have shown that while BIK is degraded by the proteasome this is not an ERK1/2‐regulated event. Modest and delayed increases in BIK following inhibition of ERK1/2 signalling actually reflect *de novo* transcription and are mimicked by inhibitors of cyclin‐dependent kinase 4 (CDK4) and CDK6 that do not inhibit ERK1/2 signalling 68. On balance it seems likely that BIK expression is cell cycle‐regulated and increases as a consequence of the G1 cell‐cycle arrest arising from inhibition of ERK1/2 signalling rather than being a direct target of ERK1/2.

In contrast to the preceding examples, the BH3‐only protein NOXA is very clearly induced rather than repressed by ERK1/2 signalling. For example, activation of ERK1/2 by activated mutant HRAS drives NOXA mRNA and protein expression, whereas inhibition of ERK1/2 signalling in tumour cells with pathway deregulation, such as BRAF^600E^ in melanoma, reduces NOXA levels 69, 70, 71, 72, 73. In terms of mechanism it appears that ERK1/2 signalling drives *NOXA* transcription via CREB; a CREB‐binding site in the *NOXA* 5′‐UTR was required for BRAF^600E^‐driven expression of a *NOXA*‐driven reporter construct while CREB phosphorylation was commensurate with NOXA expression 72. Why NOXA should exhibit this opposing reciprocal regulation by ERK1/2 signalling is unclear but it may be relevant to the onset of autophagy in response to oncogene activation, including during oncogene‐induced senescence 70. Strong ERK1/2 signalling induced by conditional overexpression of mutant HRAS increased NOXA expression, which bound to MCL1, thereby displacing Beclin‐1 leading to the activation of autophagy 70 (Fig. 3). Subsequent studies have provided support for this model by showing that NOXA is required for low‐level constitutive autophagy driven by BRAF^600E^‐MEK1/2‐ERK1/2 signalling in melanoma cells 72. Indeed, NOXA was even required for optimal starvation‐induced autophagy of melanoma cells and the authors speculated that a dynamic balance between MCL1/NOXA and MCL1/Beclin‐1 complexes may regulate ‘autophagy or apoptosis’ decisions during nutrient starvation, with ERK‐driven expression of NOXA favouring autophagy to facilitate tumour cell survival under nutrient‐poor conditions 72. In addition, ERK‐dependent up‐regulation of NOXA by mutant KRAS is also able to sensitise premalignant human epithelial cells to the combination of a SMAC mimetic (SM83) and camptothecin (CPT). However, this effect on NOXA and sensitisation to SM83/CPT is lost in malignant colorectal cancer cells with KRAS mutations due to aberrant activation of PI3K‐dependent survival signalling 73. This effect was attributed to PKB/AKT activation using the tricyclic nucleoside Triciribine, an inhibitor of DNA synthesis that has also shown potent activity against PKB/AKT, but was not confirmed using PKB/AKT‐specific RNAi.

In summary, the ERK1/2 pathway has emerged as major regulator of the mitochondrial BCL2‐regulated apoptotic pathway through the regulation of multiple prosurvival BCL2 proteins (Fig. 2) and prodeath BOPs (Fig. 3). In recent years, efforts have turned to taking advantage of this regulation to drive cell death as a therapeutic approach in cancers with deregulated ERK1/2 signalling.

#### Leveraging ERK1/2 pathway addiction with BH3‐mimetics to drive tumour cell death

Tumour cells with BRAF or KRAS mutations that drive strong activation of ERK1/2 typically become ‘addicted’ to the pathway for proliferation or survival. However, although ERK1/2 pathway inhibition promotes the expression of multiple BOPs 60, including BIM and PUMA (the most potent BOPs) the most common response to ERK1/2 pathway inhibition is cell cycle arrest in G1 rather than cell death. In tumour cells this failure to induce cell death leads to adaptation, the emergence of cells with acquired resistance and treatment failure. However, the BOPs induced by ERK1/2 inhibition may ‘prime’ tumour cells for death, tipping the balance towards apoptosis, by taking advantage of the therapeutic window provided by ERK1/2 pathway addiction. It is increasingly apparent that prosurvival proteins provide a strong buffering capacity against the BOPs that accumulate upon ERK1/2 inhibition; indeed, prosurvival BCL2 proteins are frequently up‐regulated in tumour cells 74. Thus inhibiting prosurvival BCL2 proteins is an attractive strategy to combine with ERK1/2 pathway inhibition to promote tumour cell death.

This strategy is best exemplified by ABT‐737 and ABT‐263 (navitoclax), small molecule BH3‐mimetics that inhibit BCL2, BCL‐X_L_ and BCL‐w 75, 76. In contrast to putative BH3‐mimetics such as obatoclax 77, these agents induce cell death that absolutely requires BAX or BAK 78; furthermore, both molecules displace proapoptotic proteins from BCL2, BCL‐X_L_ and BCL‐w 79, indicating that they act ‘on target’. Notably, ABT‐737 and ABT‐263 have low affinity for MCL1 and A1/BFL1 and are therefore effective at killing cells addicted to high levels of BCL2 and BCL‐X_L_. Indeed, high levels of MCL1 or A1 are associated with intrinsic 78, 80 and acquired resistance to ABT‐737 81. ABT‐263 is orally bioavailable and has shown activity in the treatment of chronic lymphocytic leukaemia (CLL) 82, 83; results in solid tumours have been more modest, perhaps reflecting higher levels of, and dependence upon, MCL1 or A1.

Several studies have now shown that combining an ERK1/2 pathway inhibitor with ABT‐737 or ABT‐263 drives apoptosis. The first report showed that MEK1/2 inhibitors U0126 and PD0325901 combined synergistically with ABT‐737 to induce tumour cell death 84. Subsequent studies showed that the MEK1/2 inhibitor selumetinib synergised with ABT‐263 to kill *BRAF*‐ and *RAS*‐mutant colorectal cancer and melanoma cell lines, causing a striking inhibition of long‐term clonogenic survival 60, 85. The BRAF‐selective inhibitor PLX4720 also combined with ABT‐737/263 in BRAF‐mutant cancer cells 85. Combined MEK1/2 and BCL2/BCL‐X_L_ inhibition is also effective in *KRAS*‐mutant lung and pancreatic tumour cells 86, 87 and acts ‘on target’ since cell death was caspase‐dependent, required BAX and BAK 60 and was confined to tumour cells addicted to ERK1/2 signalling 60, 84, 86, 88. ERK1/2 pathway inhibitors also synergise with ABT‐737 to kill acute myeloid leukaemia cells 89. Combining a MEK1/2 inhibitor with ABT‐263 is also effective *in vivo,* including in xenograft models of the *KRAS*‐mutant colorectal cancer cell lines (HCT116, SW620, SW1463) and a genetically engineered KRAS‐driven lung cancer mouse model where the combination caused 70–80% tumour regression 86.

There is clearly a role for BIM in tumour cell death arising from combined ERK1/2 pathway and BCL2/BCL‐X_L_ inhibition, but this varies across tumour type and with oncogenic driver mutation. In the BRAF^V600E^‐positive COLO205 colorectal cancer cell line, the combination of MEK1/2 inhibitor with ABT‐263 strongly induced the expression of BIM and BMF and BIM knockdown reduced apoptosis by 50–70% 60. Similarly, knockdown of BIM in BRAF^V600E^‐positive melanoma cell lines provided 50–70% protection against the combination of PLX4720 and ABT‐263 85. However, in HCT116 cells (KRAS^G13D^) MEK1/2 inhibition strongly induced BIM, BMF and PUMA expression, but knockdown of BIM and/or PUMA did not inhibit apoptosis induced by selumetinib and ABT‐263 60.

Immunoprecipitation studies have confirmed that ABT‐263 acts ‘on target’ to elicit apoptosis in combination with ERK1/2 pathway inhibitors. For example, in COLO205 cells and HCT116 cells, selumetinib promoted accumulation of BIM and BMF and their binding to both BCL‐X_L_ and MCL1 60, whereas combination with ABT‐263 caused BIM and BMF to redistribute from BCL‐X_L_ to MCL1. In HCT116 cells the combination also promoted the redistribution of PUMA on to MCL1 60. Thus, while ABT‐263 can inhibit BCL‐X_L_, but not MCL1, combination with ERK1/2 pathway inhibitors promotes the redistribution of BIM, BMF and PUMA from BCL‐X_L_ to MCL1, resulting in greater inhibition of the prosurvival BCL2 proteins and far greater cell death.

Combining ERK1/2 pathway inhibitors with ABT‐263 not only provides superior primary efficacy but also delays the onset of acquired resistance. For example, *KRAS*‐ and *BRAF*‐mutant tumour cells rapidly adapt and develop 100‐fold resistance to selumetinib 90. In contrast, treating colorectal cancer cells with selumetinib plus ABT‐263, either continuously or for as little as 3 days, inhibited the frequency of colonies that developed acquired resistance to selumetinib by 90–95% 60. This is consistent with the sustained durable tumour regressions seen with selumetinib plus ABT‐263 in *KRAS*‐mutant colorectal xenograft models and *KRAS*‐driven lung cancer genetically engineered mouse models 86. These studies exemplify the success of combining ERK1/2 pathway inhibitors BCL2 and BCL‐X_L_ inhibitors in ERK1/2 addicted tumour cells.

In addition to BCL2 and BCL‐X_L_, MCL1 is an important target for cancer therapy. The *MCL1* gene is frequently amplified in human cancers and MCL1 has been shown to be required for the survival of diverse tumour types 91, 92, 93, 94. MCL1 expression is driven by various oncogenic signalling cascades and confers intrinsic and acquired resistance to BCL2/BCL‐X_L_ antagonists and other chemotherapies 70, 80, 81. However, the development of small molecule MCL1 inhibitors has lagged behind that of BCL2/BCL‐X_L_ inhibitors. The P2 and P4 pockets of the MCL1 BH3‐binding groove differ from the equivalent pockets in BCL‐X_L_ and/or BCL2: in MCL1 P2 is more open and structurally rigid compared to P2 in BCL‐X_L_, which exhibits substantial plasticity upon ligand binding; in MCL1 P4 is poorly defined and more solvent exposed compared to BCL‐X_L_ 95, 96. These structural differences explain why ABT‐263 and ABT‐737 have very low affinity for MCL1, and why developing small molecules to directly and potently inhibit MCL1 has been challenging.

Although numerous MCL1 antagonists have been reported, including S1 and its derivatives, maritoclax (marinopyrrole A) and UMI‐77, these compounds exhibit modest affinity, insufficient selectivity, little cellular activity and/or kill cells in a BAK/BAX‐independent manner 97, 98. Improved affinity and selectivity for MCL1 over other prosurvival BCL2 proteins was more recently demonstrated with the small molecule A‐1210477 99. However, this compound binds strongly to serum proteins resulting in modest cellular potency in MCL1‐dependent cell lines and pharmacological properties, making it unsuitable for translation *in vivo* 99.

The recently described MCL1 inhibitor, S63845, provides the long sought‐after breakthrough required to robustly target MCL1 *in vivo* 100. S63845 binds with high affinity to the BH3‐binding groove of MCL1, and selectively induced apoptosis of MCL1‐dependent tumour cells in a BAK/BAX‐dependent manner with ~ 1000‐fold greater potency than A‐1210477. S63845 was well tolerated *in* v*ivo* at efficacious doses, with activity against human multiple myeloma and AML xenografts, and strikingly cured 70% of mice transplanted with Eμ‐Myc mouse lymphomas 100. Although efficacy of the monotherapy in solid tumours was modest, S63845 combined with targeted kinase inhibitors, including ERK1/2 pathway inhibitors, to kill some solid tumour cell lines *in vitro*.

The option to now robustly antagonise MCL1, in addition to BCL2 (venetoclax (ABT‐199)) and BCL2/BCL‐XL/BCL‐w (navitoclax (ABT‐263)), is likely to become a crucial weapon in the armoury against cancer, potentially as a monotherapy in haematological diseases and in combination with other targeted agents in solid tumours. Indeed, one putative MCL1 inhibitor, AMG176, for which the structure has yet to be disclosed, is already entering phase I clinical trials for patients with multiple myeloma (ClinicalTrials.gov identifier NCT02675452).

In summary, oncogene addiction provides a therapeutic window for tumour selective anticancer agents exemplified by the success of BRAF and MEK inhibitors in melanoma 10, 11. The cell response to these agents is typically cytostatic allowing tumour cells to quickly adapt and acquire resistance; however, ERK1/2 inhibition increases the expression of multiple proapoptotic BOPs, thereby sensitising them to BH3‐mimetics targeting BCL‐X_L_ and/or BCL2 or MCL1. This synthetic lethality effectively harnesses ERK1/2 inhibition, transforming cytostatic responses into cell death, improving primary efficacy and delaying the onset of acquired resistance to ERK1/2 pathway inhibitors.

### Regulation of mitochondrial fission by ERK1/2 signalling

**Figure 4:**
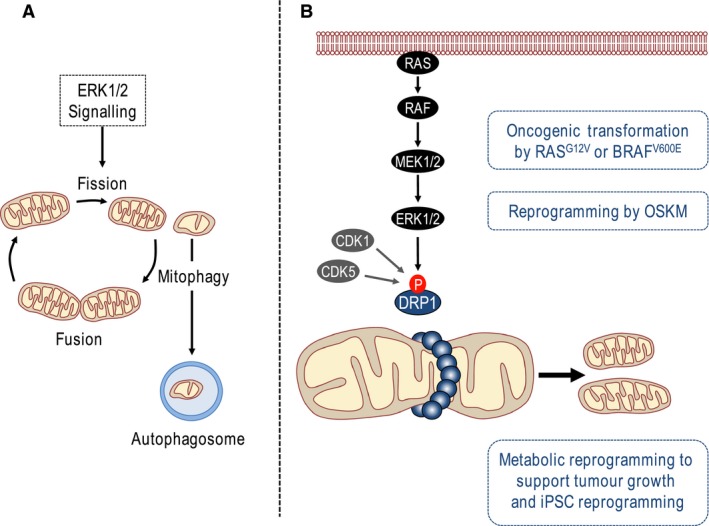
Regulation of DRP1 and mitochondrial fission by ERK1/2 signalling. (A) Cycles of fission and fusion help the mitochondrial network to adapt to the changing metabolic needs of the cell and allow the intermixing and spreading of metabolites, enzymes and mitochondrial DNA throughout the entire mitochondrial network. Mitochondrial fission also provides a mechanism for segregating dysfunctional mitochondria that can then be cleared by mitophagy as part of a fusion–fission–mitophagy quality control pathway. ERK1/2 signalling promotes mitochondrial fission. (B) The large GTPase dynamin‐related protein 1 (DRP1) is recruited to sites of mitochondrial constriction where it forms a higher order ring structure that promotes mitotic fission via GTP‐dependent scission. Phosphorylation of DRP1 at Ser616 by CDK1 or CDK5 reverses its sequestration at microtubules, promoting mitochondrial translocation of DRP1 and consequent mitochondrial fission/fragmentation. Recently it has been shown that ERK1/2 also phosphorylates DRP1 at Ser616 to promote mitochondrial fission/fragmentation as part of the metabolic reprograming that underpins RAS‐ or BRAF‐drive tumourigenesis and iPSC reprogramming following expression of the four stemness transcription factors Oct4, Sox2, Klf4 and c‐Myc (OSKM).

Mitochondrial dysfunction is a hallmark of aged cells and many age‐related diseases including forms of metabolic disease, dementia and cancer. The accumulation of dysfunctional mitochondria can cause oxidative stress, oxidative cellular damage and impaired cell function so mitochondrial ‘quality control’ is critical for lifelong health. Mitochondria are highly dynamic organelles that undergo cycles of fission and fusion which help the mitochondrial network to adapt to the changing metabolic needs of the cell; fusion allows the intermixing and spreading of metabolites, enzymes and mitochondrial DNA throughout the entire mitochondrial network, whilst fission ensures the equal segregation of mitochondria during mitosis and mitochondrial replication 101. Cycles of fusion/fission are also intimately involved in the removal of damaged or dysfunctional mitochondria (Fig. 4A). Fission is associated with major changes in the mitochondrial membrane potential (∆ψ_m_) and generates functionally divergent daughter mitochondria that can differ in their ∆ψ_m_ by up to 5 mV 102. Furthermore, since mitochondrial fusion requires an intact ∆ψ_m_ 103, these daughter cells differ in their ability to undergo a subsequent fusion event; for example, depolarized mitochondria generated during fission are far less likely to be involved in a consecutive fusion event than their functional sister mitochondria 102. This provides a mechanism for segregating nonfusing dysfunctional mitochondria that can then be cleared by mitophagy as part of a fusion–fission–mitophagy quality control pathway 104 (Fig. 4A).

Several studies have previously implicated ERK1/2 signalling in mitophagy. For example, activated mutants of MEK or ERK2 were shown to promote mitophagy with the activated ERK2 mutant actually colocating with mitochondria 105 while knockdown of ERK2 or inhibition of ERK1/2 signalling with the pan‐MEK inhibitor, U0126, blocked both starvation‐ and hypoxia‐induced mitophagy 16. Given the link between mitochondrial fission and mitophagy it is interesting that several studies have recently suggested a role for ERK1/2 signalling in the control of mitochondrial fission and implicated this in pathological mitochondrial morphology in Alzheimer's disease (AD), in the promotion of tumour growth and even reprograming of induced pluripotent stem cells (iPSCs).

Mitochondrial fission is promoted by the large GTPase dynamin‐related protein 1 (DRP1) 106. Activation of DRP1 results in its increased interaction with mitochondrial outer membrane receptor proteins, including mitochondrial fission factor (Mff), mitochondrial fission protein 1 (Fis1) and mitochondrial elongation factor 1 and 2 (MiD51 and MiD49) 107, 108. Activated DRP1 is recruited to sites of mitochondrial constriction where it forms a higher order ring structure that allows for mitotic fission to occur via GTP‐dependent scission (Fig. 4B). Another dynamin family GTPase, Optic Atrophy 1 (OPA1) cooperates with the mitofusins (MFN1 and MFN2) to promote mitochondrial fusion 109.

#### ERK1/2‐dependent regulation of MFN1

Scorrano and colleagues found that activation of ERK1/2 signalling promoted mitochondrial fission/fragmentation 110. For example, a constitutively active MEK1 mutant could promote mitochondrial fragmentation and this was defective in MFN1‐/‐ mouse embryo fibroblasts (MEFs). Conversely, dominant negative MEK1 promoted mitochondrial elongation and this was also defective in MFN1‐/‐ MEFs. So MFN1 was apparently required for ERK1/2 signalling to exert any influence on mitochondrial morphology. They used mass spectrometry to investigate whether exogenous Flag‐tagged MFN1 or MFN2 were phosphorylated and identified Thr562 of MFN1 as a phosphorylation site. Through elegant mixing experiments the authors were able to show that wild‐type and Thr562Ala MFN1 were able to form high‐molecular weight oligomers, whereas a putative phosphomimetic Thr562Asp mutant was recovered in low molecular weight fractions suggesting that phosphorylation at Thr562 might inhibit the pro‐fusion function of MFN1. They also showed that Thr562, a proline‐directed site (Thr562‐Pro) was phosphorylated by ERK1/2 *in vitro* and that phosphorylation at Thr562 reduced MFN1 oligomerisation; this in turn was argued to promote a modest increase in mitochondrial fragmentation, BAK oligomerisation and increased susceptibility to apoptotic stimuli. However, a concern with this study was the evidence that Thr562 was actually an ERK1/2 phosphorylation site *in vivo*. For example, while partially purified MFN1 was phosphorylated by recombinant ERK *in vitro*, and this was apparently reduced in the Thr562Ala mutant, a phospho‐Thr562 specific antibody showed poor reactivity even with overexpressed MFN1. In addition, phospho‐Thr562 detected with this antibody exhibited very weak increases in response to classical ERK1/2 agonists. Thus, while ERK1/2 signalling could clearly promote mitochondrial fission/fragmentation and Thr562 of MFN1 may be an important regulatory site controlling the pro‐fusion activity of MFN1, evidence for direct regulation of MFN1 by ERK1/2 was less compelling.

#### ERK1/2‐dependent regulation of DRP1

Dynamin‐related GTPase protein 1 is known to be regulated through post‐translational modifications, including phosphorylation 111. Phosphorylation at Ser616, by at least two distinct kinases, has previously been shown to promote DRP1 recruitment to the mitochondrial membrane and induce mitochondrial fission, CDK1 was shown to phosphorylate Ser585 of rat DRP1 (equivalent to Ser616 in human) during mitosis, 112; this is consistent with Ser616 residing within a proline‐directed motif (Ser616‐Pro) and CDK1 being a proline‐directed kinase. Subsequently, CDK1‐dependent phosphorylation of Ser616 was shown to reverse the sequestration of DRP1 at microtubules, promoting mitochondrial translocation of DRP1 and consequent mitochondrial fission/fragmentation 113. More recently, two studies have argued that CDK5 also phosphorylates DRP1 at Ser616 in postmitotic neurons, but with contrasting effects on DRP1 function. Cho and coworkers demonstrated that CDK5‐mediated phosphorylation promoted the dissociation of DRP1 oligomers into monomers, thereby attenuating the fission‐promoting activity of DRP1 114. Conversely, Jahani‐Asl *et al*. 115 showed that CDK5‐dependent phosphorylation of rat DRP1 at Ser585 (Ser616) increased mitochondrial fragmentation. The underlying reasons for the apparently contradictory conclusions of these two studies are unclear, but on balance the evidence to date indicates that CDK1 or CDK5‐dependent phosphorylation of DRP1 at Ser616 probably promotes mitochondrial fission/fragmentation.

In addition to CDK1 and CDK5 several studies have now suggested a role for ERK1/2 in regulating DRP1 function. Yu *et al*. 116 demonstrated that ERK1 could phosphorylate DRP1 *in vitro* and in cells this appeared to require an intact Ser616 site but the functional consequences were not assessed. Gan *et al*. 117 studied oxidative stress responses in cytoplasmic hybrid (Cybrid) derivatives of SH‐SY5Y neuronal cells, which incorporated platelet mitochondria from AD or age‐matched non‐AD human subjects. They showed that oxidative stress‐mediated ERK1/2 activation increased DRP1 expression, augmented DRP1 recruitment to mitochondria and shifted mitochondrial dynamics towards excessive pathological fission in AD cybrids. Whilst inhibition of ERK1/2 signalling with pan‐MEK inhibitors protected against the increase in mitochondrial fission, no direct functional link between ERK and DRP1 was defined 117.

However, two recent studies have now demonstrated that Ser616 of DRP1 is phosphorylated by ERK1/2 in cancer cells and that this promotes mitochondrial fission to support RAS‐dependent transformation and tumour growth 118, 119. Serasinghe and coworkers transformed primary MEFs by infection with E1A and oncogenic RAS^G12V^ and noted a marked increase in mitochondrial fission, compared to uninfected controls 118. This was accompanied by an increase in DRP1 mRNA and protein expression and DRP1 was found to be required for E1A+RAS^G12V^‐induced transformation. Furthermore, RAS^G12V^ drove MEK‐ERK1/2‐dependent phosphorylation of DRP1 at Ser592 (the murine equivalent of Ser616) in cells and recombinant ERK1 and ERK2 phosphorylated DRP1 at this site *in vitro*. Evidence of MEK‐ERK1/2‐dependent phosphorylation of DRP1 was strong with robust phosphospecific antibody data. Inhibition of ERK1/2 signalling with MEK inhibitors resulted in the rapid loss of Ser592 phosphorylation and an increase in mitochondrial fusion, which was also observed in human cancer lines with oncogenic BRAF^V600E^ mutations. The authors argued that ERK1/2‐dependent DRP1 phosphorylation increased mitochondrial fission to support tumour cell growth but this in turn conferred a vulnerability such that cells with phosphomimetic Ser616Asp DRP1 mutant became addicted to ERK1/2 signalling and underwent apoptosis upon ERK1/2 inhibition.

An independent study 119 also showed that HRAS^G12V^ promoted DRP1‐dependent mitochondrial fragmentation in SV40/hTERT‐immortalised HEK cells. Furthermore, shRNA‐dependent knockdown of DRP1 strongly inhibited the growth of these HRAS^G12V^ transformed cells as tumour xenografts. The authors showed convincingly that ERK2 can phosphorylate human DRP1 at Ser616 *in vitro*, while cells expressing activated RAS, RAF or MEK mutants exhibited strong phosphorylation of Ser616 that was abolished by MEK inhibitors. MEK inhibitors also reversed the mitochondrial fission/fragmentation observed in cells expressing activated RAS, RAF or MEK although no formal link was established between Ser616 phosphorylation and the effects of ERK signalling on mitochondrial morphology. Finally, pancreatic cancer cell lines exhibited MEK‐dependent DRP1 phosphorylation and mitochondrial fragmentation, and the growth of BxPC3 pancreatic cancer cells as tumour xenografts was inhibited by DRP1 shRNA. Taken together these two studies strongly suggest that ERK1/2 signalling can drive mitochondrial fission/fragmentation and that ERK1/2‐dependent DRP1 phosphorylation plays a prominent role in this remodelling of mitochondrial dynamics. They also suggest that some tumour cells may become addicted to ERK1/2‐dependent DRP1 phosphorylation and that DRP1 inhibition might be a potential therapeutic strategy for such tumours.

Finally, ERK1/2‐dependent mitochondrial fission/fragmentation and phosphorylation of DRP1 has recently been described during the reprogramming of somatic cells to induced pluripotent stem cells (iPSCs). Prior work had shown that the transduction of somatic cells such as MEFs with four stemness transcription factors (Oct4, Sox2, Klf4 and c‐Myc; abbreviated OSKM) caused their reprogramming to iPSCs and this was accompanied by striking metabolic reprogramming. This included a switch away from oxidative phosphorylation in favour of glycolysis and an increase in mitochondrial fission 120, 121, consistent with reports that embryonic stem cells rely on glycolytic ATP generation 122. Prieto *et al*. 121 went on to show that OSKM‐induced mitochondrial fission was dependent upon DRP1 and was accompanied by an increase in phosphorylation of DRP1 at the murine equivalent of human Ser616 with kinetics matching DRP1 recruitment to mitochondria. Expression of OSKM activated ERK1/2, apparently as a result of OSKM‐induced repression of the ERK phosphatase DUSP6. Finally, OSKM‐induced mitochondrial fission was inhibited by a MEK inhibitor, whereas expression of a DRP1Ser‐to‐Asp phosphomimetic mutant rescued fission in the presence of the MEK inhibitor. The authors concluded that ERK signalling was required for OSKM‐induced mitochondrial fission early in iPSC reprogramming, likely involving DRP1 phosphorylation. The evidence in support of ERK1/2 promoting mitochondrial fission early in reprogramming was strong and convincing. However, the role of ERK1/2 signalling in iPSC reprogramming *per se* is far more complicated. For example, inhibition of ERK1/2 signalling using a MEK inhibitor is part of a well established ‘2i’ protocol for maintaining a ground state of pluripotency in mouse ES cells 123. Thus, it seems that ERK1/2 signalling may perform different functions during reprogramming: early in reprogramming it promotes metabolic changes, including DRP1‐dependent mitochondrial fission, as part of a shift to glycolysis, whereas later it is involved in instructive differentiation signals which must be inhibited (for example by 2i) to stabilise the pluripotent state.

Dysregulation of mitochondrial dynamics leads to the accumulation of impaired mitochondria with age and contributes to alterations and pathology linked to ageing 124. These three studies 118, 119, 121 describe a common observation: ERK1/2‐driven, DRP1‐dependent mitochondrial fission/fragmentation, in RAS‐ or RAF‐driven cancers and during stem cell reprogramming (Fig. 4B). The parallels between cell reprogramming and tumorigenesis are increasingly recognised and in this context both processes exhibit a pronounced ‘Warburg effect’, a metabolic shift away from oxidative metabolism to glycolysis for ATP generation 120. Mitochondrial fission appears to be part of this metabolic reprogramming and these studies suggest that ERK1/2‐dependent DRP1 phosphorylation contributes to this mitochondrial fission. In the case of cancer at least DRP1 might represent a target for potential therapeutic intervention. In the case of stem cell reprogramming this may influence ‘stemness’ and the ability to mobilise stem cells in response to damage throughout life. It will be interesting to examine how ERK1/2‐dependent DRP1 regulation and mitochondrial dynamics changes with age and in other age‐related pathologies such as diabetes and neurodegeneration.

## Summary

The ERK1/2 signalling pathway has emerged as a major regulator of the cell‐intrinsic BCL2‐regulated apoptotic pathway, while recent work also suggests that ERK1/2 signalling controls mitochondrial fission/fusion. Past studies have suggested links between the BCL2 apoptotic machinery and proteins involved in mitochondrial fission/fusion 125. For example, BAX and BAK complex with the MFNs to facilitate MFN2 oligomerisation and activity in healthy cells 126, whereas BAK dissociates from MFN2 and associates with MFN1 during apoptosis 127. However, no consensus has emerged for the biological roles of these protein interactions and the role of mitochondrial morphology and fusion/fission in apoptosis is still not fully understood. For example, DRP1 stimulates BAX oligomerisation and cytochrome c release, two classical markers of apoptosis; however, this effect is actually independent of the canonical GTPase function of DRP1 that is required for promoting mitochondrial fission 128. More recently, downregulation of DRP‐1 was shown to prevent mitochondrial fragmentation and reduce the extent of apoptosis induced by the combination of an MCL1 inhibitor and BCL‐X_L_ inhibitor; however, combined silencing of BCL‐X_L_ and MCL‐1 resulted in extensive apoptosis without mitochondrial fragmentation indicating that apoptosis and mitochondrial fragmentation are dissociable 129. DRP1 knockdown also partially reduced apoptosis in cells in which BCL‐X_L_ and MCL1 were knocked down, suggesting that protection against apoptosis afforded by loss of DRP1 was independent of mitochondrial fragmentation. The authors speculated that the requirement for DRP1 in cell death might reflect its effects on MOMP. However, since changes in ∆ψ_m_ can disrupt mitochondrial fusion and thereby influence mitochondrial homeostasis *per se* it is difficult to be sure if the partial requirement for DRP1 in apoptosis is a direct effect or more simply reflects a decline in mitochondrial function which in turn undermines apoptotic signalling at the mitochondria. Teasing these scenarios apart will prove challenging but informative. Several studies have also argued for so‐called ‘moonlighting’ functions for BCL2 proteins in the control of mitochondrial function and aspects of metabolism distinct from their role in apoptosis 130, 131. These include BCL2, BCL‐X_L_, MCL1, NOXA, BIM and BAD, all of which are regulated at one level or another by ERK1/2 signalling. The rapid localisation of active ERK1/2 at mitochondria, their association with and/or regulation of mitochondrial proteins including BCL2 proteins, VDAC and DRP1 exemplifies the effects of ERK1/2 signalling on cell survival and mitochondrial dynamics. Based on this, future studies should take a more holistic view of ERK1/2 signalling at the mitochondria and its role in controlling cell fate decisions and disease, guided by new developments in spatial subcellular proteomics 132. Such approaches are likely to discover new roles for ERK1/2, and new substrates, at the mitochondria and other organelles.

## Conflict of interest

MS's salary was paid by AstraZeneca. SC is a consultant for Astex Pharmaceuticals but no other author received payment or consultancy.

## Author contributions

SC defined the scope of the review, wrote the introduction and summary, contributed to specific sections, prepared the final version and figures. All other authors contributed to specific sections of the review and contributed to the content of the figures.
